# The expression of spinal methyl-CpG-binding protein 2, DNA methyltransferases and histone deacetylases is modulated in persistent pain states

**DOI:** 10.1186/1744-8069-8-14

**Published:** 2012-02-27

**Authors:** Keri K Tochiki, Joel Cunningham, Stephen P Hunt, Sandrine M Géranton

**Affiliations:** 1 Department of Cell and Developmental Biology, University College London, London WC1E 6BT, UK

**Keywords:** MeCP2, Astrocyte, Microglia, Spinal nerve injury, Inflammation, Chronic pain, DNA methyltransferase, Histone deacetylase

## Abstract

**Background:**

DNA CpG methylation is carried out by DNA methyltransferases and induces chromatin remodeling and gene silencing through a transcription repressor complex comprising the methyl-CpG-binding protein 2 (MeCP2) and a subset of histone deacetylases. Recently, we have found that MeCP2 activity had a crucial role in the pattern of gene expression seen in the superficial dorsal horn rapidly after injection of Complete Freund's Adjuvant (CFA) in the rat ankle joint. The aim of the present study was to analyse the changes in expression of MeCP2, DNA methyltransferases and a subset of histone deacetylases in the superficial dorsal horn during the maintenance phase of persistent pain states. In this process, the cell specific expression of MeCP2 was also investigated.

**Results:**

Using immunohistochemistry, we found that neurones, oligodendrocytes and astrocytes expressed MeCP2. Microglia, oligodendrocyte precursor cells and Schwann cells never showed any positive stain for MeCP2. Quantitative analyses showed that MeCP2 expression was increased in the superficial dorsal horn 7 days following CFA injection in the ankle joint but decreased 7 days following spared nerve injury. Overall, the expression of DNA methyltransferases and a subset of histone deacetylases followed the same pattern of expression. However, there were no significant changes in the expression of the MeCP2 targets that we had previously shown are regulated in the early time points following CFA injection in the ankle joint. Finally, the expression of MeCP2 was also down regulated in damaged dorsal root ganglion neurones following spared nerve injury.

**Conclusion:**

Our results strongly suggest that changes in chromatin compaction, regulated by the binding of MeCP2 complexes to methylated DNA, are involved in the modulation of gene expression in the superficial dorsal horn and dorsal root ganglia during the maintenance of persistent pain states.

## Background

The induction and maintenance of persistent pain states, whether inflammatory or neuropathic, involve a number of changes in gene expression both in the dorsal root ganglia (DRGs) [1-4] and in the spinal cord [5-7]. Regulation of gene expression can be achieved through DNA CpG methylation, a process implemented by DNA methyltransferases (DNMTs). DNA methylation induces chromatin remodeling and gene silencing through a transcriptional repressor complex comprising the Methyl-CpG-binding protein 2 (MeCP2) and a subset of histone deacetylases (HDACs) [8]. Recently, we have found that MeCP2 activity had a crucial role in the pattern of gene expression seen in the superficial dorsal horn following injection of Complete Freund's Adjuvant (CFA) into the rat ankle joint [5]. A rapid increase in expression of a family of genes under the transcriptional control of MeCP2 occurred following CFA injection. These included the serum- and glucocorticoid- regulated kinase (SGK1), FK 506 binding protein 5 (FKBP5), a glucocorticoid receptor-regulating co-chaperone of hsp-90, and the sulfotransferase family 1A, phenol-preferring, member 1 (SULT1A1). Crucially, we found that SGK1 supported the induction of ankle joint inflammation indicating a key role for MeCP2 controlled epigenetic mechanisms in the induction of persistent pain states [5].

Patterns of DNA methylation are established and maintained by DNMTs. Although this process has generally been regarded as a fairly stable event in the adult animal, recent evidence suggests that it is dynamically regulated in the adult nervous system and that this may be crucial for synaptic plasticity and memory formation [9]. Since pain processing and memory formation share a number of mechanisms and are both dependent on synaptic plasticity, it is likely that DNA methylation and therefore DNMT activity is regulated during the development of long-term pain states. HDAC expression is similarly likely to change in long-term pain states. Indeed, HDACs contribute to chromatin compaction by removing acetyl groups on histone tails allowing for interaction with the DNA backbone. Others have shown that a subset of HDACs were regulated during the short-term thermal hyperalgesia that develops after injection of CFA into the hindpaw [10]. Here, we focused our interest on specific HDAC isoforms, namely HDAC 1 and 2, known to be part of the MeCP2 repressor complex [8] and HDAC 5, the isoform exhibiting the greatest change in the spinal cord after injection of CFA in the hindpaw [10].

In the present study, we analysed the expression levels of MeCP2, DNMTs and HDACs in two models of persistent pain states (ankle joint inflammation induced by CFA [5] and spared nerve injury, SNI [11,12]). Changes in mRNA expression were analysed 7 days after the initial insult, a time point when mechanical hyperalgesia is stable. We additionally investigated the cell specificity of MeCP2 expression in the superficial dorsal horn. The role of MeCP2 in the induction of pain states has so far been investigated in neurones [5,13], but recent studies have shown that MeCP2 was expressed in subsets of glial cells [14-18]. Considering the key role of glia in the development of chronic pain states [19,20], specifically astrocytes [21-23] and microglia [24-26], the potential role for glial MeCP2 needs to be examined.

## Results

### Cell specific expression of MeCP2 in the superficial dorsal horn

**Figure 1:**
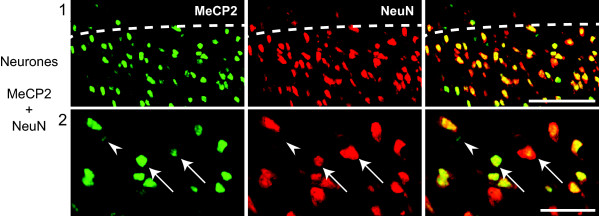
**All neurones express MeCP2 in the rat superficial dorsal horn**. Confocal images of rat superficial dorsal horn sections. Colocalization of MeCP2 (*green*; Millipore antibody) and NeuN (*red*). MeCP2 can be seen within the nucleus of all neurones (arrows). However, some MeCP2 staining is clearly non-neuronal (arrow heads). Pictures show single focal plane. Scale bars, 1) 50 μm and 2) 20 μm.

**Figure 2:**
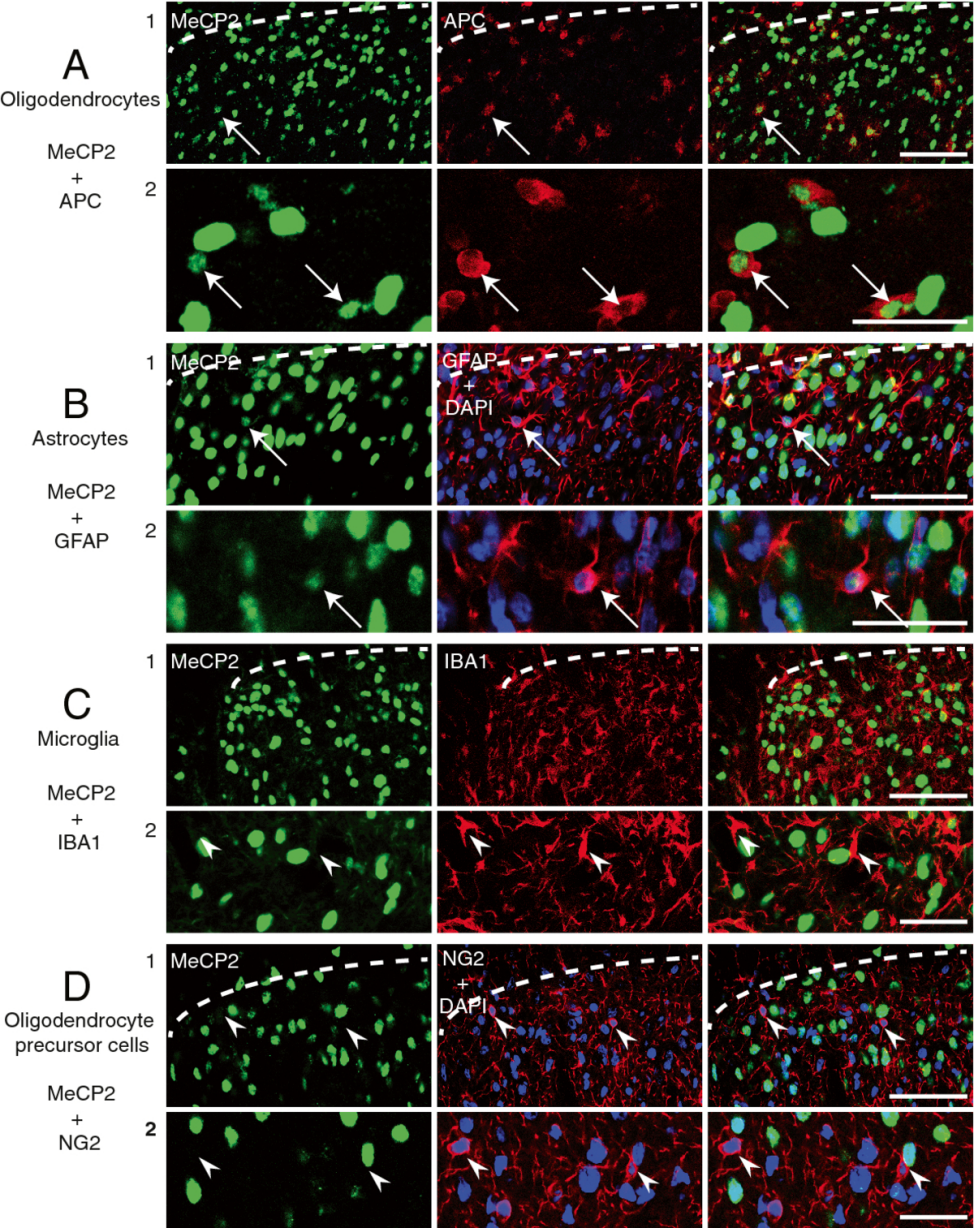
**MeCP2 is expressed in oligodendrocytes and astrocytes, but not in microglia or oligodendrocyte precursor cells, in the rat superficial dorsal horn**. Confocal images of rat superficial dorsal horn sections. ***A***, Colocalization of MeCP2 (*green*; Millipore antibody) and APC (*red*). MeCP2 can be seen within the nucleus of oligodendrocytes. ***B***, Colocalization of MeCP2 (*green*; Millipore antibody), GFAP (*red*) and DAPI, a nuclear marker (*blue*). MeCP2 can be seen within the nucleus of astrocytes. ***C***, Expression of MeCP2 (*green*; Millipore antibody) and IBA1 (*red*). MeCP2 stain cannot be seen within microglia. ***D***, Expression of MeCP2 (*green*; Millipore antibody), NG2 (*red*) and DAPI, a nuclear marker (*blue*). MeCP2 stain cannot be seen within OPCs. Pictures show single focal plane. Scale bars, 1) 50 μm and 2) 20 μm.

The neuronal and glial expression of MeCP2 was investigated in the superficial dorsal horn of animals that had undergone SNI surgery 7 days before perfusion, animals that had received a CFA injection in the ankle joint 7 days before perfusion and sham animals for both groups. While all neurones (anti-neuronal nuclei (NeuN) positive cells) expressed MeCP2 in all conditions (Figure 1), expression of MeCP2 in glial cells was at lower levels. Oligodendrocytes (anti-Adenomatosis Polyposis Coli (APC, CC-1) positive cells; Figure 2A) and astrocytes (anti-Glial Fibrillary Acidic Protein (GFAP) positive cells; Figure 2B) expressed MeCP2 only when saturating the signal from neuronal MeCP2. However, microglia (anti-Ionized calcium-binding adaptor molecule 1 (IBa1) positive cells) and oligodendrocyte precursor cells (OPCs; NG2 positive cells) never showed any MeCP2 stain (Figure 2C and 2D, respectively).

### MeCP2 expression increases in the superficial dorsal horn 7 days post CFA injection in the ankle joint

**Figure 3:**
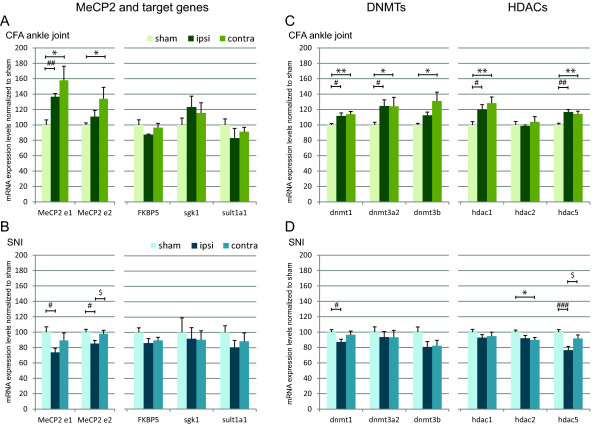
**Expression of MeCP2, DNMTs and HDACs increases within the dorsal horn 7 days following CFA injection in the ankle joint but decreases 7 days following SNI surgery**. The expression of MeCP2, downstream targets, DNMTs and HDACs was quantified by RT-qPCR. Y axis represents mRNA expression as a percentage of control (sham animals). Data show mean ± SEM. *: *P *< 0.05 and ** *P *< 0.01 sham vs contra; # *P *< 0.05, ## *P *< 0.01 and ###: *P *< 0.001 sham vs ipsi; $ *P *< 0.05 ipsi vs contra. CFA: N = 5 in each group and SNI: N = 7 in each group.

MeCP2 expression in the superficial dorsal horn was analysed using RT-qPCR. MeCP2 isoforms e1 and e2 are generated by alternative splicing of exon 2 to produce proteins with differing N termini [27]. Both MeCP2 transcripts are expressed almost ubiquitously with higher expression of the e1 isoform in the brain [28]. MeCP2 isoforms were investigated using specific primers [29]. We found a bilateral increase in MeCP2 e1 but a unilateral increase of MeCP2 e2 in the contralateral side 7 days post CFA injection (Figure 3A and Table 1). We then investigated the expression of the genes that we had found were under MeCP2 control in the superficial dorsal horn shortly after CFA injection into the ankle joint. We found no significant change in SGK1, FKBP5 and SULT1a1 expression (Figure 3A and Table 1).

**Table 1:** Primers sequence for RT-qPCR and results of RT-qPCR analysis.

mRNA		Primers sequence	CFA		SNI	
			Ispi vs Sham	Contra vs Sham	Ispi vs Sham	Contra vs Sham
MeCP2 e1	F	5'-ggagagactggaggaaaagtca-3'	137% **	158% *	74% *	ns
	R	5'-ccttcttaaacttcaggggtttc-3'				
MeCP2 e2	F	5'-ctgtttgggagaagcagagg-3'	ns	134% *	85% *	ns
	R	5'-tggtagctgggatgttaggg-3'				
FKBP5	F	5'-caccctgagcctggagagag-3'	ns	ns	ns	ns
	R	5'-gtgtcgccattactcgcagag-3'				
SGK1	F	5'-gggctgtcttgtatgagatgc-3'	ns	ns	ns	ns
	R	5'-gtgccttgctgagttggtg-3'				
SULT1a1	F	5'-ccgaggagactgtggattc-3'	ns	ns	ns	ns
	R	5'-gcatagtgggcatcaaagc-3'				
DNMT1	F	5'-ggagtgtgtgaaagagaaa-3'	112% *	114% **	87% *	ns
	R	5'-tagccttcctcagacaat-3'				
DNMT3a	F	5'-aattgtgtcttggtggat-3'	125% *	124% *	ns	ns
	R	5'-actgagaatttgccatct-3'				
DNMT3b	F	5'-ggagacagcagacatctt-3'	ns	131% *	ns	ns
	R	5'-gtcactacagttcccattaac-3'				
HDAC1	F	5'-cctcaccgaatccgaatg-3'	120% *	128% **	ns	ns
	R	5'-cgaatagaacgcaagaacttg-3'				
HDAC2	F	5'-tcaagtttctacgatcaataagg-3'	ns	ns	ns	90% *
	R	5'-cttctccgacattaaatctctg-3'				
HDAC5	F	5'-aagtacgttcaaggctaa-3'	117% **	114% **	77% ***	ns
	R	5'-cgactgctctcttcttaa-3'				
β-Actin	F	5'-agattactgccctggctccta-3'	ns	ns	ns	ns
	R	5'-aggatagagccaccaatccac-3'				
HGPRT	F	5'-aggacctctcgaagtgttggatac-3'	ns	ns	ns	ns
	R	5'-tgtagattcaacttgccgctgtc-3'				

* *P *< 0.05; ** *P *< 0.01; ns: non significant. CFA N = 5 in each group and SNI N = 7 in each group

### MeCP2 expression decreases in the superficial dorsal horn 7 days post SNI surgery

Both MeCP2 e1 and e2 decreased ipsilaterally following SNI surgery but again there were no significant changes in the expression of MeCP2 target genes (Figure 3B and Table 1).

### The expression levels of DNMTs and HDACs in the superficial dorsal horn parallel that of MeCP2 7 days post CFA injection in the ankle joint or SNI surgery

Changes in MeCP2 expression in the superficial dorsal horn are likely to be an indication of changes in chromatin compaction. We asked whether the expression levels of DNMT1, DNMT3a and DNMT3b and that of a subset of HDACs (HDAC1, 2 and 5) were also modulated during persistent pain states. We found that the expression pattern of DNMTs and HDACs mirrored that of MeCP2 with an overall bilateral up-regulation 7 days following injection of CFA in the ankle joint and ipsilateral downregulation 7 days following SNI surgery (Figure 3C and 3D respectively, and Table 1).

### MeCP2 expression in DRG neurones 7 days post SNI surgery

**Figure 4:**
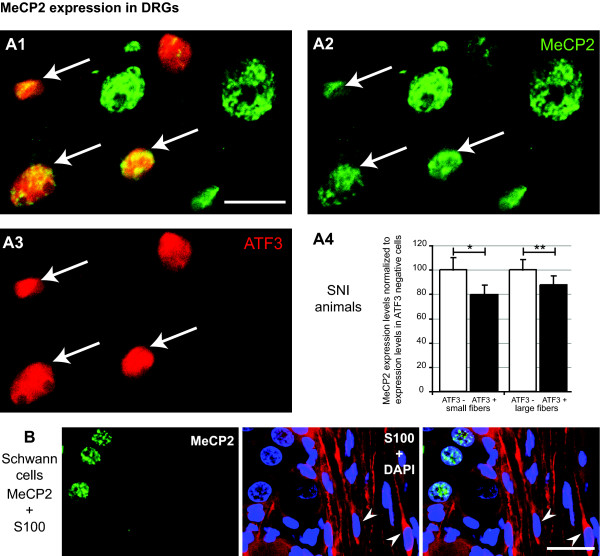
**MeCP2 expression is reduced within damaged DRG neurons following SNI surgery**. ***A1 **to **A3***, Images of DRG sections 7 days following SNI surgery. Colocalization of MeCP2 (*green*; Millipore antibody) and ATF3 (*red*) in DRG neurones. Arrow indicates neurones expressing both MeCP2 and ATF3. Scale bar, 20 μm. ***A4***, Measure of MeCP2 expression in ATF3 positive neurons using confocal microscopy. MeCP2 expression was quantified by measuring staining intensity and normalized to MeCP2 expression in ATF3 negative cells within each sub-group (small and large fibers; cell body diameter < 25 μm and > 25 μm). ***B***, Confocal images of rat dorsal root ganglion sections. Expression of MeCP2 (*green*; Millipore antibody), S100 (*red*) and DAPI, a nuclear marker (*blue*). Pictures show single focal plane. Scale bars, 50 μm.

Since peripheral nerve damage (SNI) was shown to reduce MeCP2 expression in the dorsal horn, we wanted to know what the effect of nerve transection would be on MeCP2 neuronal levels in DRGs. Our hypothesis was that MeCP2 would be decreased in injured neurones allowing for up-regulation of gene expression required for regeneration. MeCP2 expression was analysed in DRG sections stained for ATF3, a marker for damaged neurones, 7 days following SNI surgery (Figure 1, 2, 3). We measured MeCP2 expression in 4 to 5 DRG sections per animal, 1 to 9 cells per section in 4 animals. We found that MeCP2 expression in both small and large fibers was decreased in ATF3 positive neurones when compared with ATF3 negative neurones (100 ± 10.1 vs 79.4 ± 8.5 in small fibers, *P *< 0.05, and 100 ± 8.4 vs 87.6 ± 7.4 in large fibers, *P *< 0.01; Figure 4A4). Finally, since MeCP2 was expressed in subsets of glial cells in the superficial dorsal horn, we analysed MeCP2 expression in Schwann cells and found that Schwann cells (S100 positive cells) did not express MeCP2 (Figure 4B).

## Discussion

In the present study, we showed that the mRNA levels of MeCP2, DNMTs and a subset of HDACs were modulated in the superficial dorsal horn during persistent pain states, suggesting that the state of chromatin compaction is regulated during the maintenance phase of chronic pain states. We also looked at the cell specificity of MeCP2 expression and found that MeCP2 was expressed in neurones and in non-neuronal cells, specifically astrocytes and oligodendrocytes, but at much lower levels. However the changes in MeCP2 expression were measured by RT-qPCR and therefore not attributable to a specific cell type.

### Cell specific expression of MeCP2

The cell specificity of MeCP2 expression is a source of controversy [30]. While a number of studies have found that MeCP2 was not expressed in glial cells [28,31-34], more recent studies clearly showed MeCP2 expression in astrocytes, OPCs, oligodendrocytes [14,15,18,35] and microglia [14,16]. Authors of these studies suggested that the discrepancies with previous reports were due to the use of different primary antibodies and different immunohistochemical methodologies. Clearly the choice of the antibody is of crucial importance and we have found that some anti-MeCP2 antibodies can indeed lead to false positive staining (see Methods section). In the present study, we have stained rat spinal cord tissue with a commercial antibody (Millipore) successfully used by others to show MeCP2 expression in glial cells in mouse brain tissue [15,16]. We have also applied a TSA amplification method which can reveal the expression of a protein at a very low level. Under these conditions, we found that MeCP2 was expressed in astrocytes and oligodendrocytes but at much lower levels than in neurones. However, we failed to confirm the expression of MeCP2 by OPCs and microglia. Considering that others have clearly shown that the level of MeCP2 in microglia in the brain is even lower than that in astrocytes [16], it could be that the level of expression of MeCP2 in microglia in the spinal cord is not detectable in the present conditions. MeCP2 expression levels have indeed been reported to show important regional variation [34] and therefore this could also explain why we could not find MeCP2 within OPCs but in oligodendrocytes while others have reported a similar level of expression for MeCP2 in both cell types in the brain [14].

### Changes in MeCP2, DNMT and HDAC expression during the maintenance phase of persistent pain states

Regardless of the cell type, while we did not observe any significant changes in MeCP2 phosphorylation in the spinal cord 7 days following injection of CFA in the ankle joint or SNI surgery (data not shown), we found some significant changes in the level of expression of MeCP2. While MeCP2 was up-regulated in the model of inflammation, there was a clear downregulation in the neuropathic model. These changes were paralleled by that of DNMTs and a subset of HDACs. Patterns of DNA methylation are established and maintained by DNMTs. While DNMT1 is the maintenance DNMT that ensures that methylation patterns are copied faithfully throughout each cell division, DNMT3a and DNMT3b, regulate *de novo *DNA methylation in response to environmental factors. An up-regulation of DNMT expression together with an up-regulation of MeCP2 and a subset of HDACs would suggest an increase in chromatin compaction and therefore an overall downregulation of gene expression. Indeed, we found in a previous microarray study that there was a majority of down regulated genes in the superficial dorsal horn 7 days following CFA injection in the ankle joint. Remarkably, we have also shown that 2-6 h after CFA injection in the ankle joint gene transcription is globally upregulated while the expression of MeCP2, DNMTs and the same subsets of HDACs is decreased [5,36].

The downregulation of MeCP2, DNMTs and HDACs 7 days following SNI should also indicate an overall up-regulation of gene expression in the maintenance phase of this persistent pain model. A number of microarray studies have looked at changes at gene expression in neuropathic pain models but many have focused on DRGs and only few have investigated the superficial dorsal horn and classified both up and down regulated genes [7,37]. However, it was shown that there are a majority of up-regulated genes 7 days following L5 transection which would support our present finding [7].

Remarkably the changes in epigenetic mediators in the superficial dorsal horn were bilateral in our model of joint inflammation. This was not surprising since many studies report contralateral effects in arthritis models. Although poorly understood, neurogenic mechanisms are believed to be central to the development and maintenance of these contralateral effects [38]. Specifically, in the model of CFA induced joint inflammation, symmetry of joint involvement is seen after 2 to 4 weeks [39,40] while contralateral sensory nerves show altered function prior to evident signs of inflammation [41]. These observations could explain the bilateral changes in epigenetic mediators seen in the present study.

The changes in MeCP2 expression reported here indicate that MeCP2 is likely to be involved in the regulation of genes responsible for sustaining an increased level of hypersensitivity during the maintenance of persistent pain states. However, 7 days post CFA injection or post-SNI, there were no significant changes in the expression of the MeCP2 targets that were modulated during the onset of CFA induced ankle inflammation [5]. These results suggest that, even when the mechanical hyperalgesia has reached a steady level, the combination of genes expressed during the course of a persistent pain state is highly specific to the pain model and time point studied.

Finally, this study is the first to look at MeCP2 expression within DRGs. We found that MeCP2 levels were decreased in ATF3 positive neurones after SNI. MeCP2 therefore probably contributes to the large changes in gene expression seen in DRGs after SNI surgery as well as other neuropathic pain models [1,3,42] by permitting the expression of regeneration associated genes.

### DNMT inhibitors, HDAC inhibitors and the regulation of inflammatory pain states

Our results show that DNMT and HDAC mRNAs are up-regulated within the superficial dorsal horn 7 days following CFA injection in the ankle joint. This raises the possibility that DNMT and HDAC inhibitors might reduce the long-term increase in sensitivity induced by CFA. A previous study showed that the intrathecal injection of zebularine, a DNA methylation inhibitor that forms a covalent complex with DNA methyltransferases, reduced pain sensitivity when administered shortly before or after CFA injection in the hindpaw [43]. However the alleviation of the hypersensitivity was short lived (1 h) and zebularine was inefficient at reducing pain sensitivity when given 24 h after CFA. HDAC inhibitors have also been shown to provide analgesia in models of hindpaw inflammation [10,44] but whether these effects could be long-term is again unknown. The possibility that DNMT and HDAC inhibitors could reduce the sensitivity induced by CFA injection in the ankle joint also remain to be explored.

### MeCP2 and glia

While our data show changes in MeCP2 expression in the superficial dorsal horn, we did not determine in which cell type these changes occurred. Since the expression level of MeCP2 is much greater in neurones than in glia, we could expect the MeCP2 changes to reflect neuronal expression levels. This would be supported by our previous study that showed changes in MeCP2 phosphorylation specifically in neurones of the superficial dorsal horn 1 h after CFA injection in the ankle joint. However, these changes in MeCP2 expression levels could also be due to large changes in astrocytic MeCP2 levels. Although it was recently reported that changes in DNA methylation do not seem to occur within microglia and astrocytes [45], glial MeCP2 seem to be crucial to normal CNS functions. Rett syndrome (RTT) is an X-chromosome linked autism spectrum disorder caused by mutations in the gene encoding MeCP2. Until recently the disease was attributed to the loss of MeCP2 function in neurons [30]. However, new studies have challenged this assumption by showing that MeCP2 deficient glia causes abnormalities in neighbouring neurones, strongly suggesting that glia has a role in the progression of Rett's syndrome [14-16,46]. Considering the importance of glia in the development of chronic pain states, the role of spinal astrocytic MeCP2 in pain states requires further examination. A crucial experiment would be to find out if some astrocytic genes are regulated by MeCP2 and contribute to persistent pain states.

## Conclusions

Our results clearly show that MeCP2, DNMT and HDAC levels are modulated within the superficial dorsal horn during the maintenance phase of persistent pain states. It is therefore highly likely that changes in DNA methylation and chromatin compaction contribute to the regulation of pattern of gene expression responsible for the maintenance of long-term pain states. Finally, since MeCP2 is not only expressed in neurones but also in subsets of glial cells, it is crucial to further investigate the role of MeCP2 in glia during chronic pain states, especially in astrocytes.

## Methods

### Subjects

All procedures complied with the UK Animals (Scientific Procedures) Act 1986. Male Sprague Dawley rats (200-220 g; Central Biological Services, University College London, UK) were used for all experiments. Animals were kept in their home cages at 21°C and 55% relative humidity with a light-dark cycle of 12 h (lights on at 08:00 h). Food and water were provided ad libitum. All efforts were made to minimise animal suffering and to reduce the number of animals used.

### Antibodies and drugs

**Figure 5:**
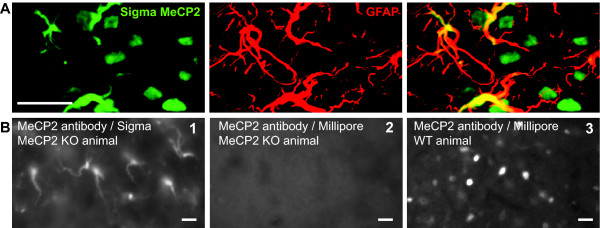
**MeCP2 antibody from Sigma wrongly labels astrocytic processes in the superficial dorsal horn**. ***A***, Colocalization of MeCP2 (*green*; Sigma antibody) and astrocytes (*red*) in the superficial dorsal horn of naïve rat. ***B***, Images of dorsal horn sections of MeCP2 knock out animals (gift from A.Bird) stained with anti-MeCP2 antibody from Sigma (1) and from Millipore (2). (3) Dorsal horn of wild type animals (C57BL/6) stained with the Millipore anti-MeCP2 antibody. Scale bar, 20 μm.

We began our study using an antibody against MeCP2 from Sigma. While we only expected a specific nuclear stain, we observed a clear labelling of astrocytic cytoplasm in the rat superficial dorsal horn (Figure 5A). In order to ascertain the validity of this stain, we used tissue from universal MeCP2 knock-out (KO) animals (gift from A. Bird) and found out that the antibody we were using was also labelling astrocytic-like processes in the dorsal horn of MeCP2 KO mice (Figure 5B1), suggesting that this was a non-specific stain. In contrast, the antibody directed against MeCP2 purchased from Millipore only showed a specific nuclear stain in wild-type animals (Figure 5B2 and 5B3). This antibody was therefore used for the rest of this study.

Anti- MeCP2 was purchased from Sigma (Cat. No: M6818; C-terminus 741-486) and Millipore (Cat. No: 07-013; C-terminus 465-478). Anti-NG2 was obtained from Millipore (Cat. No: 05-710); anti-neuronal nuclei (NeuN) from Chemicon (Cat. No: MAB 377), anti-Glial Fibrillary Acidic Protein (GFAP) and anti-S100 from Dako (Cat. No: Z0334 and Z0311, respectively), anti-Ionized calcium-binding adaptor molecule 1 (IBa1) from Wako (Cat. No: 019-19741), anti-Adenomatosis Polyposis Coli (APC, CC-1) from Calbiochem (Cat. No: OP 80), anti- 4',6-diamidino-2-phenylindole (DAPI) from Invitrogen (Cat. No: D1306) and anti-Activating Transcription Factor 3 (ATF3) from Santa Cruz Biotechnology (Cat. No: sc-188). Complete Freund's Adjuvant (CFA) was purchased from Sigma.

### Animal preparation

#### Ankle joint inflammation

Inflammation was induced by injection of Complete Freund's Adjuvant (CFA, Sigma, Poole, UK; 10 μl) in the left ankle joint, under halothane anaesthesia induced in a closed chamber delivering 5% halothane combined with 100% O_2 _at 2 l/min and maintained during the injection by the delivery (*via *a face mask) of 1.5-2% halothane combined with 100% O_2 _(1 l/min). The needle entered the ankle joint from the anterior and lateral positions, with the ankle kept in plantaflexion to open the joint. Sham treatment consisted of anaesthetizing the animals only.

#### Spared nerve injury surgery

The spared nerve injury (SNI) was performed as described [12]. Under 2% isoflurane anaesthesia the biceps femoris muscle was exposed and sectioned to expose the sciatic nerve and its three terminal branches: the sural, common peroneal and tibial nerves. The common peroneal and tibial nerves were tightly ligated with 5.0 silk and sectioned distal to the ligation. Care was taken to avoid touching or stretching the spared sural nerve. For sham surgery, the sciatic nerve was exposed as described above but no contact was made with the nerve.

### Immunohistochemistry

For immunohistochemistry, rats were deeply anaesthetized with pentobarbital 7 days after CFA injection, SNI surgery or relevant sham surgery and perfused transcardially with saline containing 5 000 I.U./ml heparin followed by 4% paraformaldehyde (PFA) in 0.1 M phosphate buffer (PB, 250 ml per adult rat). Lumbar spinal cord and L4 and L5 dorsal root ganglia (DRGs) were dissected out, post-fixed in the same PFA solution for 2 h and transferred into a 30% sucrose solution in PB containing 0.01% azide at 4°C, for a minimum of 24 h. Spinal cords and DRGs were cut on a freezing microtome set at 40 μm.

Sections were left to incubate with anti-MeCP2 antibody for 48 h at 4°C (1: 10 000). Anti-rabbit biotinylated secondary antibody was used at a concentration of 1:400 and left on for 90 min. Samples were then incubated with avidin biotin complex (ABC Elite, Vector Lab.; 1:250 Vectastain A + 1:250 Vectastain B) for 30 min followed by a signal amplification step with biotinylated tyramide solution (Perkin Elmer, 1:75 for 7 min). Finally, sections were incubated with FITC avidin for another 2 h (1:600). For double labelling, sections were left overnight at room temperature with second primary antibody (anti-NeuN: 1:1000; anti-GFAP: 1:4000; anti-IBa1: 1:2000; anti-S100: 1:1000; anti-NG2: 1:500; anti-APC (CC-1): 1:200 and anti-ATF3: 1:200). Direct secondary was used at a concentration of 1:500 (Alexa 594). When sections were double labelled with GFAP and NG2 with also used the nuclear marker 4',6-diamidino-2-phenylindole (DAPI) as third stain. DAPI (1:10000) was added to the final PBS wash. All sections were coverslipped with Fluoromount Aqueous Mounting Medium (Sigma, Poole, UK) to protect the fluorescence from fading and stored in dark boxes at 4°C. Controls for immunohistochemistry included omitting the first or second primary antibodies. Each immunohistochemical experiment was repeated with an N of three animals minimum for each treatment and each set of double stain.

### Real time reverse transcriptase polymerase chain reaction (RT-qPCR) assay

#### Tissue collection and RNA preparation

For tissue collection, animals were terminally anaesthetized with CO2 at 7 days after CFA injections, SNI or sham surgery. The spinal cord segment corresponding to the lumbar area was rapidly removed and the ipsilateral and contralateral dorsal horn quadrant L4-L6 dissected out and frozen on dry ice. Samples were then stored at -80°C until further processing. Total RNA was extracted from homogenized dorsal quadrant using an acid phenol extraction method (TRIzol reagent, RNeasy mini-columns, Qiagen, Crawley, UK). RNA concentration was calculated from the A260 value given by the Nanodrop (Labtech International, UK). All samples showing satisfactory quality were used for RT-qPCR.

#### RT-qPCR

RNA samples were treated with DNase I (Qiagen, Crawley, UK). Equal amounts (1 μg) of total RNA were reversed transcribed using random nonamers (Sigma, Poole, UK), oligo(dT)15 primers (Promega, WI, US) and Superscript TM III RT (Invitrogen, Carlsband, CA, US), one hour at 50°C in a total reaction volume of 20 μl. cDNAs were immediately quantified by real-time PCR or kept at -20°C until further experiments. Real-time PCR reactions were performed with DNA Engine (Bio-Rad, CA) using QuantiTect SYBR Green RT-PCR Master Mix (Qiagen, Crawley, UK) with each gene-specific primers (see Table 1 for primers sequences). One μl of cDNA diluted 1/10 in H_2_0 was amplified in a three-step cycling program in a final reaction volume of 25 μl. Control cDNA samples (obtained without transcriptase) were always included, as well as samples without any cDNA template. Efficiencies of PCR were calculated for each gene using serial dilution. Reactions were performed in triplicate minimum, and threshold cycle values were normalized to β-Actin (CFA experiment) or hypoxanthine guanine phosphoribosyl transferase (HGPRT; SNI experiment) gene expression. The specificity of the products was determined by melting curve analysis. The ratio of the relative expression of target genes to β-Actin or HGPRT was calculated by using the 2ΔCT formula. Then for each gene, the expression of the ipsi and contralateral side was expressed as a percentage of the expression in sham animals (i.e. the mean expression of sham animals was arbitrarily given the value of 100% and the expression level for each single sample was re-calculated accordingly).

### Confocal microscopy

All images of double stained tissue were acquired by confocal microscopy using a laser scanning microscope (Leica TCS NT SP) as described before [47]. Post-acquisition processing was performed with Adobe Photoshop and Adobe Illustrator.

### Quantitative analysis of MeCP2 expression in DRG sections

We used confocal microscopy to measure MeCP2 expression within ATF3 positive and negative DRG neurons. Gain and laser strength were set at the beginning of this experiment and fixed throughout data collection. Representative areas of the cell population in the DRG sections were selected and scanned. Both cell bodies and their corresponding nuclei were analysed to measure MeCP2 staining intensity. The intensity of staining for each cell and nucleus was recorded in arbitrary units. The area of each cell body and nucleus was noted, as was the ATF3 staining status of the cell (positive or negative). Data from ipsilateral DRG sections were processed individually for each animal. A value for the relative total MeCP2 content for each neuronal nucleus was calculated by multiplying the mean intensity of MeCP2 immunofluorescence within each nucleus by the area of the nucleus. Each nerve cell measured was classified as either a small fibre (< 25 μm cell body diameter) or large fibre (> 25 μm) neuron and further sub-divided into groups depending on whether each cell stained ATF3 positive or negative. For each group (small and large fibres), the expression of ATF3 positive fibres was expressed as a percentage of ATF3 negative fibres (i.e. the mean expression of ATF3 negative fibre was given the value of 100% and the expression level for each nucleus re-calculated accordingly).

### Statistical data analysis

All statistical tests were performed in IBM SPSS statistics 20. To analyse qPCR data, we ran first a multivariate analysis for each pain model (all genes validated using the same samples were analysed at once) to confirm a treatment effect. The analysis was followed by an univariate test to analyse each specific gene, with LSD post-hoc analysis. To analyse MeCP2 expression in ATF3 positive neurones data we used Student's T-tests. Data were always analysed as presented in the figures.

## Abbreviations

APC: Adenomatosis Polyposis Coli; ATF3: anti-Activating Transcription Factor 3; CFA: Complete Freund's Adjuvant; DNMT: DNA methyl transferase; DRG: dorsal root ganglion; FKBP5: FK 506 binding protein 5: GFAP: Glial Fibrillary Acidic Protein; HDAC: Histone deacetylase; IBa1: anti-Ionized calcium-binding adaptor molecule 1; KO: knock-out; MeCP2; Methyl-CpG-binding protein 2; OPC: oligodendrocyte precursor cells; SGK1: serum- and glucocorticoid- regulated kinase; SNI: spared nerve injury; SULT1A1: sulfotransferase family 1A: phenol-preferring: member 1.

## Competing interests

The authors declare that they have no competing interests.

## Authors' contributions

SMG and SPH conceived, designed and performed the experiments, analysed the data. KKT and JC performed some of the experiments. All authors contributed to the writing of the manuscript and approved the final manuscript.
